# Inter- and Intra-Modal Contrastive Hybrid Learning Framework for Multimodal Abstractive Summarization

**DOI:** 10.3390/e24060764

**Published:** 2022-05-29

**Authors:** Jiangfeng Li, Zijian Zhang, Bowen Wang, Qinpei Zhao, Chenxi Zhang

**Affiliations:** 1 School of Software Engineering, Tongji University, Shanghai 201804, China; lijf@tongji.edu.cn (J.L.); qinpeizhao@tongji.edu.cn (Q.Z.); 2 Meituan-Dianping Group, Shanghai 200050, China; zijian.zh96@gmail.com

**Keywords:** multimodal abstractive summarization, cross-modal fusion, contrastive learning, supervised and unsupervised learning

## Abstract

Internet users are benefiting from technologies of abstractive summarization enabling them to view articles on the internet by reading article summaries only instead of an entire article. However, there are disadvantages to technologies for analyzing articles with texts and images due to the semantic gap between vision and language. These technologies focus more on aggregating features and neglect the heterogeneity of each modality. At the same time, the lack of consideration of intrinsic data properties within each modality and semantic information from cross-modal correlations result in the poor quality of learned representations. Therefore, we propose a novel Inter- and Intra-modal Contrastive Hybrid learning framework which learns to automatically align the multimodal information and maintains the semantic consistency of input/output flows. Moreover, ITCH can be taken as a component to make the model suitable for both supervised and unsupervised learning approaches. Experiments on two public datasets, MMS and MSMO, show that the ITCH performances are better than the current baselines.

## 1. Introduction

The last two decades have witnessed a surge of information on the internet. Extensive digital resources in a variety of formats (text, image and video) have enriched our lives, facilitated by a proportional increase in online sharing platforms, such as YouTube, Facebook, etc. Meanwhile, a large number of articles, including texts, images and videos, are continuously generated and displayed on the internet everyday. For example, BBC News provided 1.1 million multimedia articles in 2021, with 72 million daily visitors  [1].

This large amount of information provides opportunities for people to obtain what they want from the internet. However, reading such numbers of articles in their entirety is time-consuming work. Consequently, it is necessary to analyze multimedia articles and make summarizations automatically for them so that internet users can read the short summarizations rather than the whole articles.

Recently, research into multimodal abstractive summarization (MAS) has provided approaches for integrating image and text modalities into a short, concise and readable textual summary [2,3]. With the rapid development of deep learning technologies, more and more researchers have explored various methods for solving this task in unsupervised [4,5] or supervised [3,6,7] approaches. In general, the current deep-learning-based schemes are inseparable from the *extracting feature then downstream processing* [8] paradigm.

**Figure 1:**
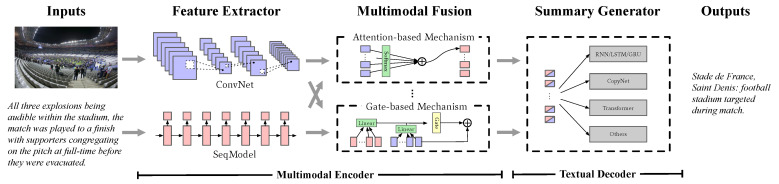
Illustration of the standard multimodal abstractive summarization framework, which consists of a multimodal encoder and a textual decoder. The decoder generates a target summary after extracting the visual semantic features and merging them together.

In the multimedia field, especially for MAS, there are usually three steps [8], which are (1) feature extraction, (2) multimodal fusion and (3) textual generation. Figure 1 shows details of the common multimodal abstractive summarization framework. Firstly, the step of feature extraction aims at extracting region- or token-level features from multimodal references using their own domain extractors, such as ConvNet and SeqModel for visual and textual data. Next, in the step of multimodal fusion, fusion information is obtained using cross-modal mechanisms (e.g., alignment or projection). After that, a target textual summary is generated by maximizing likelihood estimation or augmentation objectives in the step of textual generation.

Current research focuses more on processes of the multimodal fusion and textual generation steps instead of feature extraction, as the feature extractors have already been widely used in the fields of natural language processing (NLP) and computer vision (CV) and obtain good performance. In approaches of multimodal fusion, multiple inputs are fused by attention-based [9] or gate-based [3] mechanisms in order to learn a representation that is suitable for summary generation. Such solutions concentrate on aggregating features from several modalities. However, they ignore the heterogeneity of vision and language and do not consider that there are semantic gaps between images and text. In the research on textual generation, designing a novel decoder and adding objectives are two main approaches. The classic scheme employs recurrent neural network (RNN [10]) or CopyNet [11] as a backbone caused by the sequence properties of language. Recently, transformer-based pre-trained generative language models, such as UniLM [12], BART [13] and ProphetNet [14], have shown remarkable performance on generation tasks, one for the advantages of the self-attention module and the other for the large-scale corpus. Adding extra training goals can lead to better performance for driving the summary generation, whose typical goals are image–text [15] or text–text [16] matching. The recent research also explores a contrastive-based method to eliminate the gap between training and verification [17]. However, the above additional objectives focus more on the textual coherence rather than the semantical consistency of the input image and sentences. To summarize, the existing system has two flaws: (1) a visible gap between vision and language, and (2) a lack of consideration of intrinsic data properties within input–output sentences and semantic consistency among input cross-modal correlation.

To address the aforementioned problems, this paper provides an **I**nter- and In**T**ra-modal **C**ontrastive **H**ybrid (**ITCH**) learning framework for the MAS task. It adjusts three points of the vanilla transformer: it (1) uses the pre-trained language and vision models as encoders, (2) adds a cross-modal fusion module and (3) adds hybrid auxiliary contrastive objectives. The pre-trained vision transformer [18] (ViT) and BERT [19] are employed to encode image and text, respectively, to assure the unity of bi-modal information processing. For tackling flaw 1, we propose a cross-modal fusion module to compensate for the feature-level gap after obtaining the visual and textual features. Taking the textual data as query, the additional information is referenced from visual features for fusion. For tackling, flaw 2, the whole model incorporates two additional contrastive learning objectives based on the end-to-end textual reconstruction loss: an intra-modal objective for input and output utterances, and an inter-modal objective for input image and sentences. In addition, ITCH can be taken as a component to make the model suitable for both supervised and unsupervised learning environments. Experimental results on MSMO and MMS demonstrate that ITCH outperforms previous state-of-the-art methods on the multimodal abstractive summarization task in terms of ROUGE, relevance scores and human evaluation. The main contributions of this paper are:

(1) An ITCH framework is proposed for tackling multimodal abstractive summarization in a supervised approach. Moreover, with ITCH as a component and integrated into an existing system, it is appropriate for unsupervised learning environments.
(2) A cross-modal fusion module is designed for obtaining textually enhanced representation. It merges contextual vision and language information, and makes visual features align to textual representation.
(3) The objectives of the inter-modal and intra-modal frameworks are integrated with a reconstruction objective in summary generation. The inter-modal objective measures consistency for input images and texts, while the intra-modal objective maintains the semantic similarity between input sentences and output summary.

The rest of this paper is organized as follows: Section 2 discusses related work. Section 3 presents the ITCH framework. ITCH-based components used for supervised and unsupervised learning environments are also introduced in this section. Section 4 evaluates the performance of the ITCH framework and discusses the results. A case study is shown in Section 5. Section 6 concludes the paper.

## 2. Related Work

### 2.1. Visual and Semantic Feature Extractors

The feature extractors utilized in NLP and CV differ due to the different properties of text and images. A recurrent neural network (RNN [10]) was proposed to model sequential sentences and represent contextual features. With the increase in sentence length, the gradient dispersion limits its further development. Long short-term memory (LSTM [20]) and gated recurrent unit (GRU [21]) with a gate mechanism can help with this issue, but the technique of encoding tokens (in sentences) one at a time restricts inference efficiency. To address the above problems, transformer [22] with self-attention is proposed to contextualize the entire sentence or paragraph in features in a parallel manner. This facilitates the development of a pre-trained language model which designs specific tasks on a large-scale corpus for training. In a variety of downstream tasks, pre-trained language models such as ELMo [23], GPT [24], BERT [19] and RoBERTa [25] have achieved state-of-the-art performance. As a result, the current schemes rely heavily on the pre-trained model as a linguistic feature extractor.

For vision, a convolutional neural network (CNN [26]) is the most extensively used deep learning model. It aggregates local spatial features using a kernel and accumulates them with feedforward networks. Moreover, some studies focus on the salient regions of objects or entities using Faster R-CNN [27] in conjunction with ResNet [28] to learn features with rich semantic meaning. To connect the domains of vision and language, Dosovitskiy et al. [18] try to employ a vanilla transformer with patch projection for vision problems.

### 2.2. Multimodal Fusion Methods

Multimodal fusion is intended to fuse heterogeneous information in order to better interpret multimodal inputs and apply them to downstream tasks. The early fusion (EF [29]) aims at embedding features by projection or concatenation. Considering that EF does not accumulate intra-modal information, Zadeh et al. [30] utilizes a memory fusion network to account for modal-specific and cross-modal interactions continuously. The hierarchical attention for fusion is also proposed for addressing multimodal interaction, which was advised by Kronecker [31]. Similarly, using an attention-based mechanism, Pruthi et al. [32] apply a masked strategy for “deceiving”, which improves the attention’s reliability. Different from focusing on the information across modalities by attention, some studies have tried to fuse multimodal information from the correlation between input and output. Liu et al. [33] employ low-rank tensors of several representations, including output, to perform multimodal fusion. Furthermore, Liu et al. [34] propose a novel TupleInfo to encourage learning to examine the correspondences of input and output in the same tuple, ensuring that weak modalities are not ignored. Recently, a channel-exchanging-network (CEN [35]) was proposed for tackling the inadequacy in balancing the trade-off between inter-modal fusion and intra-modal processing.

### 2.3. Methods for Abstractive Summarization

Multimodal summarization is the task of generating a target summary based on multimedia references. The most significant difference between multimodal summarization and textual summarization is whether the input contains two or more modalities. Based on the distinct methodologies, the multimodal summarization can be separated into multimodal abstractive summarizing and multimodal extractive summarization. The former is consistent with our research, which gathers information from multiple sources and constructs textual sequences using a generation model.

For the MAS task, Evangelopoulos et al. [36] detect the key frames in a movie based on the salience of individual elements for aural, visual and linguistic representations. Replacing frames with tokens in sentence, Li et al. [37] generate a summary from a set of asynchronous documents, images, audios and videos by maximizing the coverage. Sanabria et al. [38] use a multimedia topic model to identify the representative textual and visual samples individually, and then produce a comprehensive summary. Considering visual information as a complement to textual features for generation [7], Zhu et al. [39] propose a multimodal input and multimodal output dataset, as well as an attention model to generate a summary through a text-guided mechanism. The model Select [40] proposes a selective gate module for integrating reciprocal relationships, including a global image descriptor, activation grids and object proposals. Modeling the correlation among inputs is the core point of MAS. Zhu et al. [41] frame a unified model for unsupervised graph-based summarization that does not require manually annotated document–summary pairs. Another unsupervised method which is significantly related to our paper is the generation with the *“long-short-long”* paradigm [5] combined with multimodal fusion.

### 2.4. Contrastive Learning

Much research utilizes contrastive objectives for instance comparison (gathering similar samples while keeping the distance between dissimilar samples as large as possible) in order to facilitate representation learning in both NLP and CV. For example, noise-contrastive estimation (NCE [42]) is proposed to tackle the computational challenges imposed by the large number of instance classes. Information NCE (infoNCE [43]) maximizes a lower bound on mutual information between images and caption words in cross-modal retrieval. For vision with contrastive learning, MoCo [44] further improves such a scheme by storing representations from a momentum encoder dynamically, and MoCov2 [45] borrows the multi-layer perceptron and shows significant improvements over MoCo. SimCLR [46] proposes a simple framework for large-batch applications that do not require memory representations. For language, ConSERT [47] notices that the native-derived sentence representations are proved to be collapsed in semantic textual similarity tasks. Gao et al. [48] find that dropout acts as minimal data augmentation can achieve state-of-the-art performance by utilizing a contrastive learner. For cross-modal scenarios, vision–language pre-trained methods are representatives that embrace multi-modal information for reasoning [49,50]. Recently, Yuan et al. [51] utilized the NCE [42] and MIL-NCE [52] losses to learn representations using across-image and text modalities.

## 3. The ITCH Framework

**Figure 2:**
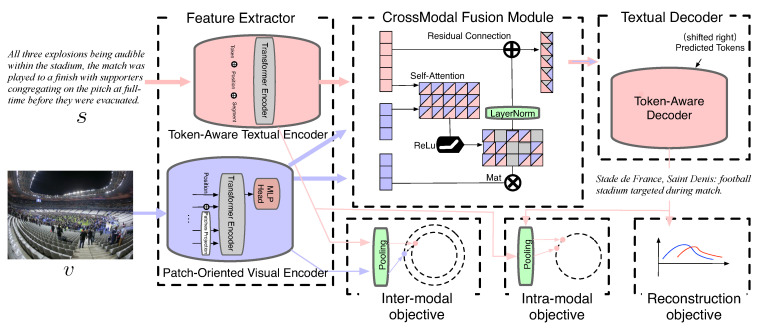
Schematic illustration of the ITCH framework with supervised learning. It comprises four components. (1) *Feature Extractor*: the visual and textual features are embedded by their own domain encoders, respectively, i.e., ViT and BERT. (2) *Cross-Modal Fusion Module*: the self-attention mechanism with ReLu and residual connection. (3) *Textual Decoder*: a traditional transformer-based decoder is used to reconstruct a summary. (4) *Hybrid Contrastive Objectives*: apart from using the common reconstruction loss for summary generation, an inter-modal contrastive objective is designed to maintain the distance among bi-modal inputs, and an intra-modal contrastive objective is used to gather information between input sentences and output utterances.

In this section, we introduce details of our proposed ITCH for a multimodal abstractive summarization task. The ITCH (illustrated in Figure 2) takes bi-modal image and text as inputs and represents their respective features using a patch-oriented visual encoder and a token-aware textual encoder in *Feature Extractor*. For the purpose of alignment, a *Cross-Modal Fusion Module* is used to enhance the semantic features. Thereafter, the target summary is generated by the token-aware decoder introduced in *Textual Decoder*. In addition, the *Hybrid Contrastive Objectives* introduces the inter- and intra-modal contrastive objectives as auxiliary objectives for the summarization referenced from multiple modalities. Finally, we also show how to use ITCH as a component for the unsupervised learning approach.

### 3.1. Visual and Textual Feature Extractor

Given a set of mini-batch input, $B={\left\{\left(s_{i},v_{i}\right)\right\}}_{i=1}^{\left|B\right|}$, where |B| is the number of examples in B. For the $i^{\mathrm{th}}$ example $\left(s_{i},v_{i}\right)$, $v_{i}\in R^{C\times H\times W}$ denotes a single image and $s_{i}\in R^{M}$ stands for sentences, where (H∈[0,255],W∈[0,255]) is the resolution of image $v_{i}$, C=3 denotes the number of channels of $v_{i}$, and *M* denotes the number of tokens in the sentences $s_{i}$. In order to represent the contextual features of images and text, respectively, different pre-trained transformer-based models were used as extractors.

**Patch-Oriented Visual Encoder**. To obtain visual features, we chose vision transformer (ViT) as extractor, which receives as input a 1D sequence of embedding, while the original image is 3D. We reshaped the image into a sequence of flattened 2D patches $v\in R^{N\times (P^{2}\cdot C)}$, where *P* is the height and width of the patches. Then, $N=HW/P^{2}$ is the resulting number of patches. Following the linear projection FC and 2D-aware position embeddings $E_{\mathrm{pos}}^{img}$, the image embeddings can feedforward to the patch-oriented visual encoder. Let *D* be the hidden dimension of ViT; the visual feature $V\in R^{N\times D}$ can then be obtained by

(1) $$V=\mathrm{ViT}\left(\mathrm{FC}\left(v\right)+E_{\mathrm{pos}}^{img}\right).$$

**Token-Aware Textual Encoder**. As for the textual branch, the pre-trained BERT is used to extract context-enhanced features. The similar operation linear projection FCs are used for token-level embedding, whose weights are not shared with the visual branch. In addition, the static positional embedding $E_{\mathrm{pos}}^{txt}$ is also considered. Following by BERT, we utilized a fully connected layer to map the same D-dimension with V. The textual feature $S\in R^{M\times D}$ is calculated as follows:

(2) $$S=\mathrm{BERT}\left(\mathrm{FC}\left(s\right)+E_{\mathrm{pos}}^{txt}\right)W_{t}+b_{t},$$

where $W_{t}$ and $b_{t}$ are trainable weights in the full-connection layer. Recall that through this section, the original image $v\in R^{C\times H\times W}$ and text $s\in R^{M}$ are represented as features $V\in R^{N\times D}$ and $S\in R^{M\times D}$.

### 3.2. Cross-Modal Fusion Module

**Figure 3:**
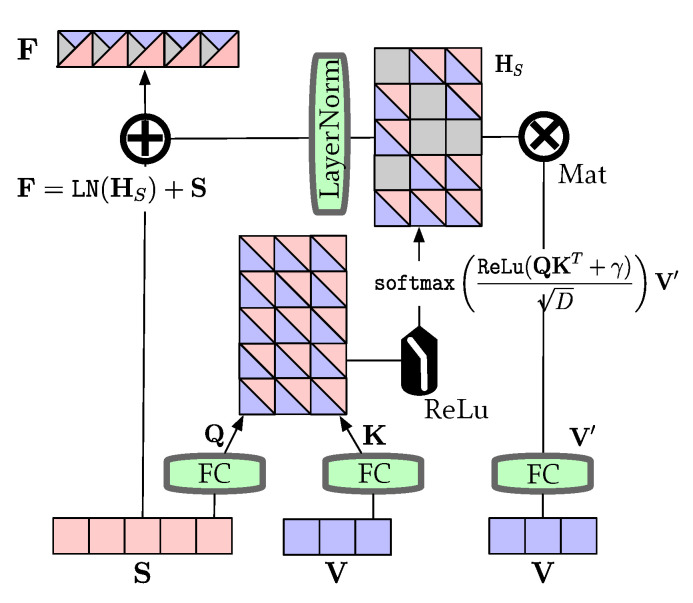
Illustration of cross-modal fusion module.

Given two encoded and unaligned features, V and S, the goal of the cross-modal fusion module is to align semantic features in S to V via query/key/value attention and modified filter (details in Figure 3). We first projected bi-modal features to vectors, i.e., $Q={\mathrm{SW}}_{Q}$, $K={\mathrm{VW}}_{K}$ and $V^{\prime }={\mathrm{VW}}_{V}$, where $W_{Q}$, $W_{K}$ and $W_{V}$ are weights. We assumed that a good way to fuse vision–language information is by providing a latent adaptation from V to S as Formula (3). In addition, an adjustable factor γ together with activation function ReLu(x)=max{x,0} was used to filter high relevance scores. That is to say, the low-value scores w.r.t unaligned visual feature are abandoned by this process. The temporary fusion feature can be presented as

(3) $$\begin{array}{cc} H_{S} & =\mathrm{softmax}\left(\frac{\mathrm{ReLu}(QK^{T}+\gamma )}{\sqrt{D}}\right)V^{\prime }\\ \; & =\mathrm{softmax}\left(\frac{\mathrm{ReLu}({\mathrm{SW}}_{Q}W_{K}^{T}V^{T}+\gamma )}{\sqrt{D}}\right){\mathrm{VW}}_{V} \end{array}.$$

Considering that the final target is a textual summary and the prevention of gradient dispersion, we utilized layer normalization [53] and residual connection [28] to enhance textual information. Then, the fusion feature $F\in R^{M\times D}$, which highlights semantic vector among vision and language features, can be calculated by

(4) $$F=\mathrm{LN}(H_{S})+S.$$

### 3.3. Textual Decoder

The goal of ITCH is to generate a target summary $\hat{Y}=\{$<sos>$,\cdots ,{\hat{y}}_{i},\cdots ,$<eos>} which begins and ends by special tokens <sos> and <eos>. The corresponding ground-truth is noted as Y. After obtaining the fusion feature $F\in R^{M\times D}$ through the cross-modal fusion module, the textual sequence is generated by a token-aware transformer-based decoder. It takes the prediction tokens ${\hat{y}}_{0:i-1}$ and fusion feature F as inputs, and outputs the current state token by model with parameters θ. In detail, the TransDec denotes the function of the decoder and the ${\hat{y}}_{0:i-1}$ means the tokens before the *i*th token, where ${\hat{y}}_{0}=$ <sos>:

(5) $${\hat{y}}_{i}=\mathrm{TransDec}\left(F,{\hat{y}}_{0:i-1}\right).$$

For the generation objective, the reconstruction loss $L_{gene}$ is taken into account naturally. It minimizes the negative log-likelihood by

(6) $$L_{gene}=-\frac{1}{\left|B\right|}\sum_{B}\sum_{i}\log p\left({\hat{y}}_{i}==y_{i}|{\hat{y}}_{0:i-1},F;\theta \right).$$

### 3.4. Hybrid Contrastive Objectives

In this section, we introduce two contrastive objectives besides the common generation objective, which can be considered auxiliary tasks during the training process that reinforce the primary summarization task. In detail, text–image consistency loss and IO (Input/Output)-aware coherence loss are proposed to maximize the lower bound on mutual information.

**Inter-modal objective for input text–image pair**. Natural matches exist between each other due to the pairing of the image and sentences in the existing datasets; although beneficial to the training process, this decreases the generalization of the models and inhibits further model performance improvements. In previous procedures, we obtained the context-enhanced visual feature V and language feature S through feature extractors. In order to facilitate the comparison of images and texts, the pooling strategy was used to abstract features into vectors.

(7) $$\left\{\begin{array}{cc} o^{v} & =\mathrm{BN}\left(\mathrm{MeanPool}(V)\right)\\ o^{s} & =\mathrm{LN}\left(\mathrm{MeanPool}(S)\right) \end{array}\right.,$$

where batch normalization BN() and layer normalization LN() are used for pooling vision and language features to vectors, respectively. Generally speaking, L2 regularization is used to map the matching to the unified space before the similarity calculation [54]. However, we did not truly want to complete the matching in our case, but tried to maintain the consistency between images and sentences. Experimental results show that using different normalization can fuse more information without destroying the distribution of data.

Following the motivation aforementioned, we expected that the corresponding image and text pair would have a high consistency, while the irrelevant pairs would have low similarity, especially those with fine-grained interplaying. To achieve this goal, we accumulated the contrastive losses advised by infoNCE directly.

(8) $$L_{inter}=-\frac{1}{\left|B\right|}\sum_{i=0}^{B}\log\frac{\exp(\mathrm{sim}(o_{i}^{s},o_{i}^{v})/\tau )}{\sum_{i\neq j}\left(\exp\left(\mathrm{sim}(o_{i}^{s},o_{i}^{v})/\tau \right)+\exp\left(\mathrm{sim}(o_{i}^{s},o_{j}^{v})/\tau \right)\right)},$$

where sim denotes the similarity function, $\mathrm{sim}\left(a,b\right)=a\cdot b^{T}$.

**Figure 4:**
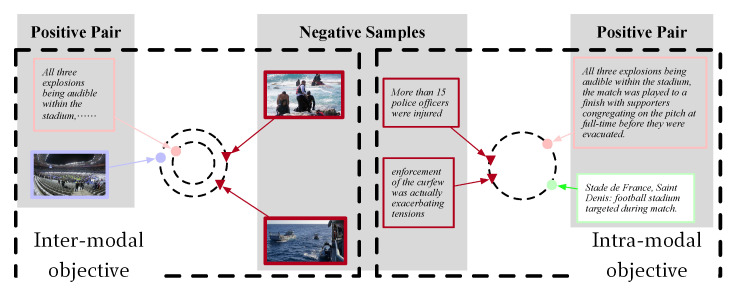
Visualization of inter- and intra-modal contrastive losses. The positive pairs in $L_{inter}$ are denoted by pink (input sentences) and blue (input image), and in $L_{intra}$ are denoted by pink (input sentences) and green (target summary) points. The negative examples are noted by red triangles.

**Intra-modal objective for input/output utterances**. The access to the coherence labels of IO utterances often requires extra expert annotations or additional algorithms, which are expensive or which may introduce error propagation. Considering the observation that sentences in reference are inherently related to the generated summary, we instead obtained the coherence by modeling the similarity of IO textual data. The assumption behind this is that utterances within the same description are more similar to one another than those spanning across different paragraphs. Similar to $L_{inter}$, the loss for measuring the coherence among IO utterances can be expressed as

(9) $$L_{intra}=-\frac{1}{\left|B\right|}\sum_{i=0}^{B}\log\frac{\exp(\mathrm{sim}(o_{i}^{s},o_{i}^{y})/\tau )}{\sum_{i\neq j}\left(\exp\left(\mathrm{sim}(o_{i}^{s},o_{i}^{y})/\tau \right)+\exp\left(\mathrm{sim}(o_{i}^{s},o_{j}^{y})/\tau \right)\right)},$$

where $o^{y}$ is the sentence embedding obtained using the same method as the textual vector $o^{s}$. We also visualized the difference between the above two contrastive losses in Figure 4.

In conclusion, the total loss function of ITCH can be defined as Formula (10), where $\left|\right|\cdot {\left|\right|}_{2}$ denotes the L2 norm for parameters θ:

(10) $$L=\sum_{B\in D}\left(L_{gene}+L_{inter}+L_{intra}\right)+{\left||\theta |\right|}_{2}.$$

### 3.5. Unsupervised Learning Combined with ITCH

**Figure 5:**
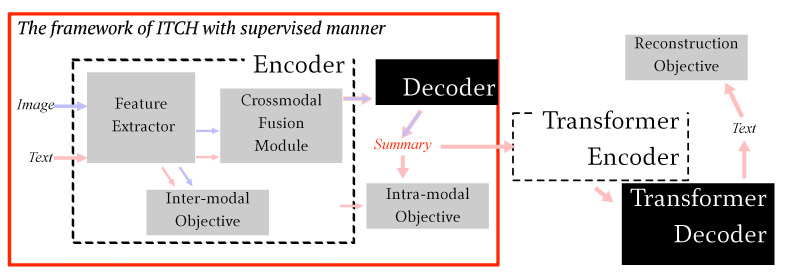
Structure for the unsupervised learning methods using the same structure of ITCH with the supervised approach as an additional component and adding a transformer model with encoder TransEnc and decoder TransDec to reconstruct the input text, which is advised for the existing Compress-then-Reconstruct approach (CTNR).

The aforementioned description is the processing flows that combine ITCH with supervised learning approach. The ITCH can easily implement unsupervised multimodal abstractive summarizing by taking the ITCH as compression. In detail for unsupervised approach, as shown in Figure 5, we utilized the existing *“long-short-long”* (CTNR [5] structure: *sentences* →Encoder-Decoder→*summary*→Encoder-Decoder→ *sentences*) structure. It fuses multimodal information and generates a summary through a decoder, and then the generated summary is taken into account for reconstructing the input sentences.

Following Section 3.1 and Section 3.2, the textual-enhanced feature F is obtained through the cross-modal fusion module. The generation processing is the same as in Equation (5). We encoded the generated summary $\hat{Y}$ and reconstructed the textual input sequences *s* because unsupervised learning cannot be trained with a corresponding label. The reconstructor is a transformer model with encoder TransEnc and decoder TransDec. The predicted input text $\hat{s}$ is calculated using the following formula:

(11) $$\begin{array}{cc} F^{\prime } & =\mathrm{TransEnc}(\hat{Y})\\ {\hat{s}}_{i} & =\mathrm{TransDec}(F^{\prime },{\hat{s}}_{0:i-1}) \end{array}.$$

The reconstruction loss of the unsupervised approach is different from that of the supervised one. The likelihood considers predicted sentences $\hat{s}$ and input text *s* rather than the generated summary $\hat{Y}$ and the ground-truth Y, while the function is the same as in Equation (6). The hybrid inter- and intra-modal contrastive losses are also considered (details in Section 3.3), and the total above processes are composed as in ITCH with the unsupervised approach.

The framework of ITCH with the supervised approach is highlighted with a red box in Figure 5 to denote the role of ITCH in the unsupervised approach. In conclusion, compared with the supervised ITCH, there are two different points in the unsupervised approach.

(1) The input and output of the whole model changes from $\left\{\left(v,s\right)\to \hat{y}\right\}$ to {(v,s)→s}. The supervised ITCH takes bi-modal inputs to generate a summary directly, while the unsupervised ITCH generates a summary in the middle of the whole model and takes these sequences to reconstruct the input text.
(2) Additional transformers, Encoder and Decoder, are added for reconstructing input sentences, while the supervised ITCH does not consider Encoder and Decoder.

## 4. Experiment

### 4.1. Setup

We evaluated the ITCH on two public multimodal summarization datasets, MMS [55] and MSMO [39]. Each sample in the MMS is a triplet (sentence, image, headline), while the headline is commonly considered a target summary. As Table 1 shows, MMS and MSMO were divided into three groups for experiments. The maximum number of words in the input sentence for the MMS dataset was 439. For the MSMO, the items are from internet news articles with numerous picture captions. After removing special tokens and punctuation, the maximum number of tokens was reduced from 740 to 492, which is applicable to the maximum length of 512 for the transformer model.

The word embedding size was set to 300 and the limited vocabulary size was set to 20,004 with four extra special tokens (<unk>, <pad>, <sos> and <eos>). The dimension *D* of feature is 768 depending on the chosen visual and language pre-trained encoders, which are advised from huggingface (*bert-based-uncased*: https://huggingface.co/bert-base-uncased, accessed on 13 April 2022, *vit-base-patch16-224*: https://huggingface.co/google/vit-base-patch16-224, accessed on 13 April 2022). We also used dropout with a probability equal to 0.3 for the cross-modal fusion module. The batch size was up to 128 limited by the GPU (Nvidia 3090 with 24 GB VRAM) and the overall parameters were trained for 30 epochs with a 2 × 10${}^{-5}$ learning rate for pre-trained extractors and 2 × 10${}^{-4}$ for others, which were halved every 10 epochs. We used mean pooling for transforming features to vectors, which has been verified as the most effective way [56] compared to Max pooling or [CLS]. For other hyperparameters, the optimal settings are: adjustable factor in cross-modal fusion module γ=−0.15 and temperature parameter in infoNCE τ=0.1. Details are shown in Table 2.

### 4.2. Evaluation Metrics and Baselines

#### 4.2.1. Evaluation Metrics

The evaluation metrics are calculated between the generated summary and the ground-truth, which judge: word-overlap, embedding relevance and human evaluation.

- **ROUGE** [57]: the standard metric to calculate the scores between the generated summary and the target sentences using the recall and precision overlaps (details are R-N and R-L). R-N refers to an N-gram recall between a candidate summary and a set of reference summaries. R-N is computed as follows:
  (12) $$R-N=\frac{\sum_{S\in \left\{ReferenceSummaries\right\}}\sum_{gram_{N}\in S}Count_{match}\left(gram_{N}\right)}{\sum_{S\in \left\{ReferenceSummaries\right\}}\sum_{gram_{N}\in S}Count\left(gram_{N}\right)},$$
  where N means the length of N-gram, and $gram_{N}$ and $Count_{match}\left(gram_{N}\right)$ are the maximum number of N-grams co-occurring in a candidate summary and a set of reference summaries. Here, we selected **R-1** and **R-2** as the evaluation metrics. **R-L** uses longest-common-subsequence (LCS)-based F-measure to estimate the similarity between two summaries. The longer the LCS of the two summaries is, the more similar the two summaries are.
- **Relevance** [58]: we used embedding-based metrics to evaluate the similarity of the generated summary and the target summary. In particular, **Embedding Average** and **Embedding Extrema** use the mean embedding and max-pooled embedding to compute the cosine similarity. **Embedding Greedy** does not pool word embeddings but greedily finds the best matching words. These metrics are used to measure the semantic similarity of the generated summary and the ground-truth.
- **Human**: we invited twelve native speakers to evaluate the generated summary according to fluency and relevancy. The judges can give a score from 0 to 4, as detailed in Table 3. We randomly sampled 100 results for each dataset and divided them into four batches. The judges were broken into four groups and each batch of samples was annotated by two groups of judges. For each sample, we used above two ratings for each aspect (fluency or relevance) and we took the average as the final rating. The male-to-female ratio was 1:1. Within a batch, if the ratings differed substantially between the two groups of judges, a third group of judges would be invited to annotate the batch. The judges did not have access to the ground-truth response, and saw only the inputs and the predicted summary.

#### 4.2.2. Baselines

In this paper, we used ITCH as a component combined with the supervised and unsupervised learning approaches for the MAS task. Therefore, the baselines were chosen as follows:

**For unsupervised learning methods**, LexRank [59] is a textual PageRank-like algorithm that selects the most salient sentences from a reference. Using embedding similarity for sorting, W2VLSTM [60] is an improvement based on LexRank. With the development of a deep neural generation network, Seq3 [4] is proposed to use the “long-short-long” pattern to automatically generate a summary. The above three methods only refer to the unimodal information to summarize utterances, while the task of an abstractive summary with reference to multimodal information is considered to be a more challenging task. Guiderank [39] is a classic method on the MSMO dataset, which is an unsupervised baseline without considering the ITCH framework. MMR [41] with SOTA performance on both MSMO and MMS uses a graph-based ranking mechanism for extraction.

**For supervised learning methods**, S2S [10] and PointerNet [61] are generation models based on Encoder–Decoder, where PointerNet can project special tokens to a target summary. With the rise in pre-trained models in the NLP field, UniLM [12] has been proven to have a strong performance in the abstractive summarization task. For a supervised framework that references multimodal information, Doubly-Attn [62] uses multiple attention modules for aggregation. MMAF and MMCF [55] are the modality-based attention mechanism for paying a different kind of attention to image patches and text units, which are filtered through selective visual information. Considering a selective gate network for reciprocal relationships between textual and multi-level visual features, SELECT [40] is the current SOTA baseline.

### 4.3. Experimental Results and Analysis

We carried out experiments to compare the performance of ITCH with baselines on the MSMO and MMS datasets in metrics: ROUGE/Relevance/Human.

**For the results and analysis on the MSMO dataset**, there were two types of experimental results, unsupervised and supervised. In terms of resource, uni-modal (uni- in Table 4) only considers textual data, while bi-modal (bi- in Table 4) contains visual and textual data as inputs, to which our method belongs. As Table 4 shows, our ITCH outperformed unsupervised and supervised competitive baselines on different metrics (ROUGE and Relevance) and created a new state of the art. Compared with the mainstream unsupervised learning model (MMR), ITCH had an average improvement of 10.67% in word-overlap-based metrics and 4.71% in embedding-based metrics; that is, $\left.\left(\sum_{\in \mathrm{ITCH}}-\sum_{\in \mathrm{MMR}}\right)/\sum_{\in \mathrm{MMR}}\right.$. The former resulted in more improvement than the latter, which indicates that the textual summary generated by unsupervised ITCH is more accurate and similar to the reference at the word-overlap level. Such a superiority benefits from our two contrastive objectives, which not only enhanced the relevance of input text and output summary but also improved the correlation of the input text–image pair.

A similar situation occurred in the comparison with the mainstream supervised learning model (Select). ITCH still performed 4.38% better in word-overlap-based metrics and 2.68% better in embedding-based metrics. This completely illustrates that our cross-modal fusion module can model and understand unaligned multimodal to reinforce the generation of a target summary. In addition, whether supervised or unsupervised, ITCH still achieved almost the highest level in human evaluation metrics considering the subjectivity. This demonstrates that the summary generated by ITCH is more readable and topic-related than other baselines. It is no exception that the performance of the unsupervised ITCH was worse than that of the supervised one because of the lack of massive manually labeled data.

**For the results and analysis on the MMS dataset**, our ITCH outperformed both unsupervised and supervised baselines. As Table 5 shows, ITCH with a unsupervised learning model exceeded all the corresponding baselines in ROUGE, Relevance and Human evaluation metrics. In particular, our method outperformed the current state-of-the-art MMR [41] by 10.29% in the ROUGE metric and 4.98% in the Relevance metric, which also indicates the remarkable advantage of our two contrastive objectives.

Compared with the supervised mainstream methods, our ITCH still has an obvious superiority. With regards to ROUGE, ITCH surpassed MMAF [55] by 4.86% and MMCF [55] by 6.47%. For the Relevance metric, our approach was also superior to MMAF [55] and MMCF [55] by about 3.34% and 3.90%, respectively. We can conclude that the cross-modal fusion module offers an overall comprehension of several modalities to improve the relevance and similarity of the summary and the inputs under the supervised condition.

### 4.4. Ablation Analysis

In this section, we analyze the roles that different factors play in the ITCH framework. There were three aspects studied on the MSMO dataset for the ablation analysis: hyperparameters, the cross-modal fusion module and hybrid contrastive losses.

A prerequisite for a summary to help users accurately acquire information is that the image be related to the target summary. Therefore, an image–text relevance metric is used to measure the quality of the generated summary and the effect of contrastive losses, which is advised by Zhu et al. [39]. The proposed metric M${}_{\mathrm{sim}}\in \left[-1,1\right]$ considers visual-semantic embedding to calculate cosine similarity between normalized visual and textual features.

**The effect of the hyperparameters.** We tested the impact of different values of two hyperparameters γ in Formula (3) and τ in Formulas (8) and (9), respectively. γ acts as a balancer to control the value of the activation function ReLu=max{x,0} for filtering high-relevance scores. According to Table 6, we obtained the best performance under both unsupervised and supervised conditions if γ was set to −0.15. If the value was greater or less than −0.15, the performance was worse. In particular, when γ was set to a greater value than the default value, V would obtain a larger share of the fusion feature, leading to a greater drop in performance. With regards to τ, a larger value had a negative impact on the result, which may have been because the effect of the cosine similarity to the loss function was decreased. In addition, if τ was set to a smaller value than the default τ=0.1, the ROUGE and Relevance metrics became worse, while M${}_{sim}$ improved. We believe that a smaller τ, together with the loss function, facilitates the optimization process of the cosine similarity between the textual feature and the visual feature. Through the above analysis, the proper hyperparameters play a crucial part in keeping our ITCH functioning optimally.

**The effect of the cross-modal fusion module and hybrid contrastive losses.** The results of the ablation studies are shown in Table 7. If ITCH discards the cross-modal fusion module, the performance decreases obviously, whether in unsupervised or supervised approaches, compared with the original ITCH and corresponding current state-of-the-art (MMR and Select). In particular, M${}_{sim}$ was reduced by 25.41% and 16.85% in comparison to unsupervised ITCH and supervised ITCH, respectively. We conclude that the cross-modal fusion module is pivotal for the improvement of the similarity between visual feature and the textual feature. Without this module, the performance of ITCH is still close to MMR’s and even exceeds Select’s, which indicates the superiority of the additional inter- and intra-modal contrastive objectives. Similarly, when removing inter-loss, intra-loss, or both, the performance of ITCH suffered universally. Furthermore, inter-modal loss had a greater influence on the M${}_{sim}$, whether using an unsupervised or a supervised method, but intra-modal loss had a stronger influence on ROUGE and Relevance in an unsupervised setting. I/O coherence influenced the fusion feature, which led to reducing the relevance between the generated summary and the corresponding image. Furthermore, the consistency of the input text–image pair played an important role in the word overlapping and embedding similarity. Significantly, ITCH with single inter-loss or intra-loss outperformed the unsupervised baseline and the supervised baseline, which fully indicates the vital function of the extra contrastive losses.

## 5. Case Study

To further analyze the ITCH framework and compare it with the baselines, we listed a series of results about a case from the MMS dataset in Table 8. A news article with numerous sentences and one image is provided as input. The text mainly states that Singapore suffers from Zika virus and dengue virus, and that the government has introduced many measures to prevent the virus from spreading. The corresponding image depicts that a firefighter is misting insecticide indoors. The output in Table 8 contains the target summary for the inputs and the summaries generated by approaches of the baselines and the ITCH in the unsupervised and supervised approaches.

For the unsupervised approach, the generated summary of our ITCH has the highest coherence with the target summary. Compared with the uni-model LexRank, the ITCH covers all salient information from the input text and image. Two types of words for “virus” and the obvious symptoms of the disease and their preventive measures appeared in the summary generated by ITCH. This demonstrates that our cross-modal fusion module fully utilizes textual and visual information from references. Moreover, the structure of ITCH’s result is the most consistent compared with the structures of generated summaries from unsupervised baselines. This reflects that our ITCH model learns the capacity of narrative logic. It is worth noting that on three metrics, R-1/R-2/R-L, ITCH was superior to both unsupervised and supervised methods. In comparison with the ground-truth, it performed poorly with advanced vocabulary and grammar. For example, the result could not recognize uncommon or complex words such as “Zika” or “mosquito-borne”.

Our ITCH generates a more thorough and readable summary that is significantly closer to the ground-truth summary when using a supervised approach. The result contains more important information compared with the unsupervised result, such as the phrases “aggressive spraying”, “indoor spraying” and “transmission”. Unlike the supervised baseline Select, which ignored information from the first paragraph of the input text, our result took into account all portions of the text and reflected the influence of the I/O contrastive loss. Although ITCH behaves as the state-of-the-art technique in the unsupervised and supervised fields, there is still space to improve, such as for unknown words.

## 6. Conclusions

In this paper, we propose the inter- and intra-modal contrastive hybrid (ITCH) learning framework, which learns to automatically align multimodal information and maintains the semantic consistency of input/output flows. We evaluated our framework with unsupervised and supervised approaches on two benchmarks (i.e., MSMO and MMS datasets) for three metrics: ROUGE, Relevance and Human Evaluation. The experimental results on all datasets show that our ITCH consistently outperforms comparable methods, whether with supervised baselines or unsupervised baselines. We further carried out comprehensive ablation studies to confirm that the proper hyperparameters, the cross-modal fusion module and hybrid contrastive losses are essential in ITCH. Furthermore, we showed a successful example from the MMS dataset to provide a more intuitive comparison. In the future, we will improve our model to better understand and summarize complicated vocabulary. Furthermore, we intend to study the multimodal abstractive summarization task on a Chinese dataset.

## Figures and Tables

**Table 1:** Dataset statistics of MMS and MSMO. Each image is paired with captions. #Train, #Valid and #Test denote the number of examples in each group. #MaxLength denotes the maximum number of tokens in captions for MMS and MSMO, respectively.

Datasets	#Train	#Valid	#Test	#MaxLength
MMS	62,000	2000	2000	439
MSMO	240,000	3000	3000	492

**Table 2:** Hyperparameters setting.

Symbol	Annotation	Value	Symbol	Annotation	Value
E	Word embedding size	300	EP	Number of epochs	30
V	Vocabulary size	30,004	DR	Dropout rate	0.3
*D*	Dimension of feature	768	γ	Adjustable factor	−0.15
|B|	Batch size	128	τ	Temperature parameter	0.1
LR0	For pre-trained modules	2 × 10${}^{-5}$	LR1	For other modules	2 × 10${}^{-4}$

**Table 3:** The metrics for the human evaluation.

Fluency (F)		Relevance (R)	
Points	Explanations	Points	Explanations
1	*Hard to read*	1	*Totally irrelevant*
2	*Not quite fluent and containing* *several grammatical errors*	2	*Marginally relevant*
3	*Fluent response with few errors*	3	*Somewhat relevant but not* *directly related to the query*
4	*Fluent response without errors*	4	*Relevant*

**Table 4:** Performance of ITCH and baselines on MSMO dataset with ROUGE/Relevance/Human. ✓ means the methods belong to either uni-modal or bi-modal. A **Bold** value means the best performance.

Types	Resource		Methods	ROUGE			Relevance			Human	
	uni-	bi-		R-1	R-2	R-L	Average	Extrema	Greedy	F	R
	✓		LexRank (2004) [59]	32.54	9.96	28.02	0.277	0.204	0.278	2.72	3.02
	✓		W2VLSTM (2018) [60]	29.86	13.11	27.68	0.278	0.201	0.296	2.54	2.82
Unsupervised	✓		Seq3 (2020) [4]	38.16	13.58	32.07	0.347	0.245	0.342	3.14	3.28
		✓	GuideRank (2018) [39]	37.13	15.03	36.18	0.332	0.231	0.341	3.12	3.31
		✓	CTNR (2021) [5]	40.11	16.97	39.71	0.372	0.271	0.386	3.32	3.44
		✓	MMR (2021) [41]	41.72	17.33	39.81	0.381	0.269	0.391	3.39	3.39
		✓	**ITCH**	**43.77**	**21.62**	**44.02**	**0.393**	**0.296**	**0.401**	**3.46**	**3.42**
	✓		S2S (2014) [10]	32.32	12.44	29.65	0.292	0.209	0.287	3.24	3.32
	✓		PointerNet (2017) [61]	34.62	13.72	30.05	0.339	0.267	0.352	3.21	3.41
Supervised	✓		UniLM (2020) [12]	42.32	22.04	40.03	0.443	0.308	0.438	**3.71**	3.54
		✓	Doubly-Attn (2020) [62]	41.11	21.75	39.92	0.434	0.297	0.433	3.46	3.52
		✓	Select (2020) [40]	46.25	24.68	44.02	0.466	0.331	0.471	3.62	3.59
		✓	**ITCH**	**47.78**	**25.39**	**46.82**	**0.476**	**0.342**	**0.484**	3.67	**3.63**

**Table 5:** Performance of ITCH and baselines on MMS dataset with ROUGE/Relevance/Human. Symbol “-” denotes that no ready-made results and no code are provided.

Types	Resource		Methods	ROUGE			Relevance			Human	
	uni-	bi-		R-1	R-2	R-L	Average	Extrema	Greedy	F	R
	✓		LexRank (2004) [59]	36.52	9.16	27.66	0.264	0.192	0.271	2.86	3.17
	✓		W2VLSTM (2018) [60]	29.14	9.77	28.11	0.272	0.202	0.283	2.71	2.92
Unsupervised	✓		Seq3 (2020) [4]	36.42	10.22	34.91	0.339	0.221	0.341	3.21	3.24
		✓	GuideRank (2018) [39]	35.31	10.11	33.91	0.302	0.211	0.312	3.01	3.15
		✓	CTNR (2021) [5]	39.69	13.16	39.22	0.371	0.254	0.357	3.37	3.37
		✓	MMR (2021) [41]	41.29	16.75	38.29	0.382	0.269	0.393	3.43	**3.41**
		✓	**ITCH**	**44.61**	**18.91**	**42.72**	**0.396**	**0.301**	**0.399**	**3.49**	**3.41**
	✓		S2S (2014) [10]	30.81	11.72	28.23	0.285	0.202	0.278	3.24	3.32
	✓		PointerNet (2017) [61]	35.61	14.64	33.62	0.345	0.271	0.355	3.32	3.46
Supervised	✓		UniLM (2020) [12]	41.82	20.82	39.83	0.451	0.311	0.459	**3.73**	3.57
		✓	Doubly-Attn (2020) [62]	39.82	19.72	38.21	0.438	0.302	0.431	3.44	3.54
		✓	Select (2020) [40]	45.63	23.68	42.97	0.466	0.327	0.473	3.64	3.55
		✓	MMAF (2021) [55]	47.28	24.85	44.48	0.472	0.336	0.480	-	-
		✓	MMCF (2021) [55]	46.84	24.25	43.76	0.470	0.335	0.476	-	-
		✓	**ITCH**	**48.62**	**26.73**	**46.93**	**0.487**	**0.351**	**0.493**	3.68	**3.61**

**Table 6:** The effect of the hyperparameters (γ and τ) on the MSMO dataset. (The symbol ↑ denotes that the value has been improved, and ↓ denotes that the value has decreased).

	Unsupervised			Supervised		
Methods	ROUGE	Relevance	M${}_{\mathrm{sim}}$	ROUGE	Relevance	M${}_{\mathrm{sim}}$
ITCH	36.47	0.363	0.547	39.99	0.434	0.623
Default γ=−0.15						
ITCH (γ=−0.20)	36.31 ↓	0.357 ↓	0.541 ↓	39.49 ↓	0.431 ↓	0.618 ↓
ITCH (γ=−0.10)	35.23 ↓	0.343 ↓	0.511 ↓	39.02 ↓	0.424 ↓	0.611 ↓
ITCH (γ=−0.05)	33.28 ↓	0.318 ↓	0.436 ↓	37.66 ↓	0.397 ↓	0.528 ↓
ITCH (γ=0)	31.74 ↓	0.394 ↓	0.402 ↓	36.81 ↓	0.389 ↓	0.503 ↓
Default τ=0.1						
ITCH (τ=0.01)	35.62 ↓	0.347 ↓	0.556 ↑	38.74 ↓	0.418 ↓	0.634 ↑
ITCH (τ=0.2)	35.17 ↓	0.339 ↓	0.493 ↓	37.87 ↓	0.401 ↓	0.589 ↓

**Table 7:** The effect of the cross-modal fusion module and hybrid contrastive losses. (Symbol “-*X*” denotes that module *X* is removed).

	Unsupervised				Supervised		
Methods	ROUGE	Relevance	M${}_{\mathrm{sim}}$	Methods	ROUGE	Relevance	M${}_{\mathrm{sim}}$
ITCH	36.47	0.363	0.547	ITCH	39.99	0.434	0.623
MMR	32.95	0.347	0.382	Select	38.32	0.423	0.452
- $L_{inter}$	34.18	0.359	0.389	- $L_{inter}$	38.66	0.427	0.471
- $L_{intra}$	33.71	0.350	0.443	- $L_{intra}$	38.89	0.428	0.581
- $L_{inter}\&L_{intra}$	30.11	0.336	0.301	- $L_{inter}\&L_{intra}$	36.91	0.396	0.449
- CrossFusion	32.71	0.343	0.408	- CrossFusion	38.51	0.425	0.518

**Table 8:** A case study from the MMS dataset. The references of the bi-modal inputs and the target summary are given in the top table. The summaries generated by ITCH and the baselines are shown in the bottom table, which also calculated ROUGE and M${}_{sim}$.

**Input**	Text	Zika is primarily spread by mosquitoes but can also be transmitted through unprotected sex with an infect- ed person. Almost daily downpours, average temperature of 30 degrees Celsius (86 degrees Fahrenheit), large green areas in a populated urban setting makes Singapore a hospita- ble area for mosquitoes. So Singapore is the only Asian country with active transmission of the mosquitoborne Zika virus, the US, Australia, Taiwan and South Korea have all issued alerts advising pregnant women against traveling to Singapore. Singapore is known to suffer wi- dely from dengue virus, a mosquito-borne tropical dis- ease that triggers high fevers, headaches, vomiting and skin rashes in those infected to a considerable extent and therefore may be mistaken for another. Singapore’s government has a long history of using aggressive spraying, information campaigns and heavy fines for homeowners who leave water vesse in the open, in a bid to control mosquito-borne dengue. Indoor spray, misting and oiling were conducted, and daily misting of common areas is ongoing, hundreds of specialist workers conduct island-wide inspections for mosquito breeding g- rounds, spray insecticide and clear stagnant water.		Image	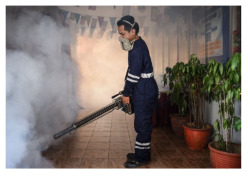		
	**Target** **Summary**	Singapore has suffered from the Zika virus and dengue virus, both of them are mosquitoborne disease with high fevers. The government employ aggressive spraying and information campaign to prevent its spread.					
	**Types**	**Methods**	**Generated Summary**	**R-1**	**R-2**	**R-L**	**M${}_{sim}$**
		LexRank	Singapore is known to suffer widely from virus, a mosquitoborne tropical disease that trigger high fevers, vomiting and skin rashes in infected.	25.8	4.0	24.1	0.36
	Unsupervised	GuidRank	Singapore is the Asian country with active transmission of <unk> virus and dengue virus. They mimic each other to a considerable extent.	29.3	11.7	20.7	0.42
		CTNR	Singapore is known to suffer from <unk>, a tropical disease that <unk> fevers in infected. It spread by and government use spread mosquitoes.	32.2	13.6	27.6	0.40
**Output**		ITCH	The Singapore in <unk> and dengue virus, and are <unk> disease with fevers. They employ spraying campaign to prevent.	48.3	25.0	44.8	0.52
		PointNet	The Singapore’s government aggressive spray and information prevent zika and dengue virus.	33.7	21.5	27.5	0.41
	Supervised	UniLM	Zika is primarily spread by <unk> and dengue virus tropical disease that triggers high fevers. They may be mistaken for another. The Singapore’s employing spraying and campaign to prevent and altering travelers.	42.0	19.7	38.1	0.44
		Select	The Singapore taking spraying and information campaign to prevent <unk> virus. It suffering from virus that are fevers and transmission.	52.1	27.9	29.6	0.51
		**ITCH**	The Singapore take aggressive spraying, indoor spraying and information campaign to prevent <unk> virus and dengue virus spread. They are <unk> disease with high fevers and transmission.	**61.3**	**42.1**	**61.0**	**0.69**

## Data Availability

The datasets (MSMO and MMS) investigated in this work are publicly available at http://www.nlpr.ia.ac.cn/cip/dataset.htm (accessed on 23 November 2021). MSMO corresponds to the “Dataset for Multimodal Summarization with Multimodal Output” proposed by conference EMNLP2018 [39]. Furthermore, MMS corresponds to the “Dataset for Multimodal Sentence Summarization” proposed by an IJCAI2018 conference paper [55].

## Abbreviations

The following abbreviations are used in this manuscript:

CNN	Convolutional neural network
CV	Computer vision
GRU	Gated recurrent unit
InfoNCE	Information NCE
ITCH	Inter- and intra-modal contrastive hybrid learning framework
LCS	Longset common subsequence
LSTM	Long short-term memory
MAS	Multimodal abstractive summarization
NCE	Noise-contrastive estimation
NLP	Natural language processing
RNN	Recurrent neural network

## References

[1] Gupta A., Chugh D., Katarya R. (2022). Automated news summarization using transformers. Sustainable Advanced Computing.

[2] Sanabria R., Caglayan O., Palaskar S., Elliott D., Barrault L. How2: A Large-scale Dataset For Multimodal Language Understanding. Proceedings of the ViGIL, NeurIPS.

[3] Liu N., Sun X., Yu H. Multistage Fusion with Forget Gate for Multimodal Summarization in Open-Domain Videos. Proceedings of the EMNLP.

[4] Baziotis C., Androutsopoulos I., Konstas I., Potamianos A. (2019). SEQ3: Differentiable Sequence-to-Sequence-to-Sequence Autoencoder for Unsupervised Abstractive Sentence Compression. Proceedings of the 2019 Conference of the North American Chapter of the Association for Computational Linguistics: Human Language Technologies, Volume 1 (Long and Short Papers).

[5] Zhang C., Zhang Z., Li J., Liu Q., Zhu H. CtnR: Compress-then-Reconstruct Approach for Multimodal Abstractive Summarization. Proceedings of the 2021 International Joint Conference on Neural Networks (IJCNN).

[6] Kato T., Matsushita M., Kando N. MuST: Workshop on MUltimodal Summarization for Trend Information. Proceedings of the NTCIR.

[7] Zhu J., Zhou Y., Zhang J., Li H., Zong C., Li C. Multimodal summarization with guidance of multimodal reference. Proceedings of the AAAI Conference on Artificial Intelligence.

[8] Chen J., Hu H., Wu H., Jiang Y., Wang C. Learning the Best Pooling Strategy for Visual Semantic Embedding. Proceedings of the IEEE Conference on Computer Vision and Pattern Recognition (CVPR).

[9] Libovický J., Helcl J. Attention Strategies for Multi-Source Sequence-to-Sequence Learning. Proceedings of the ACL.

[10] Sutskever I., Vinyals O., Le Q.V. (2014). Sequence to sequence learning with neural networks. Adv. Neural Inf. Process. Syst..

[11] Gu J., Lu Z., Li H., Li V.O. (2016). Incorporating Copying Mechanism in Sequence-to-Sequence Learning. Proceedings of the 54th Annual Meeting of the Association for Computational Linguistics, Volume 1: Long Papers.

[12] Dong L., Yang N., Wang W. (2019). Unified Language Model Pre-training for Natural Language Understanding and Generation. arXiv.

[13] Lewis M., Liu Y., Goyal N., Ghazvininejad M. BART: Denoising Sequence-to-Sequence Pre-training for Natural Language Generation, Translation, and Comprehension. Proceedings of the ACL.

[14] Qi W., Yan Y., Gong Y., Liu D. Prophetnet: Predicting future n-gram for sequence-to-sequence pre-training. Proceedings of the EMNLP: Findings.

[15] Tu R.C., Ji L., Luo H., Shi B., Huang H., Duan N., Mao X.L. (2021). Hashing based Efficient Inference for Image-Text Matching. Proceedings of the Findings of the Association for Computational Linguistics: ACL-IJCNLP 2021.

[16] Zhong M., Liu P., Chen Y., Wang D., Qiu X., Huang X. (2020). Extractive Summarization as Text Matching. Proceedings of the 58th Annual Meeting of the Association for Computational Linguistics.

[17] Liu Y., Liu P. SimCLS: A Simple Framework for Contrastive Learning of Abstractive Summarization. Proceedings of the ACL/IJCNLP.

[18] Dosovitskiy A., Beyer L., Kolesnikov A., Weissenborn D., Zhai X., Unterthiner T., Dehghani M., Minderer M., Heigold G., Gelly S. (2021). An Image is Worth 16×16 Words: Transformers for Image Recognition at Scale. arXiv.

[19] Devlin J., Chang M.W., Lee K., Toutanova K. (2019). BERT: Pre-training of Deep Bidirectional Transformers for Language Understanding. Proceedings of the 2019 Conference of the North American Chapter of the Association for Computational Linguistics: Human Language Technologies, Volume 1 (Long and Short Papers).

[20] Hochreiter S., Schmidhuber J. (1997). Long short-term memory. Neural Comput..

[21] Cho K., van Merriënboer B., Gulcehre C., Bahdanau D., Bougares F., Schwenk H., Bengio Y. (2014). Learning Phrase Representations using RNN Encoder–Decoder for Statistical Machine Translation. Proceedings of the 2014 Conference on Empirical Methods in Natural Language Processing (EMNLP).

[22] Vaswani A., Shazeer N., Parmar N., Uszkoreit J., Jones L., Gomez A.N., Kaiser L., Polosukhin I. (2017). Attention is all you need. Advances in Neural Information Processing Systems.

[23] Peters M.E., Neumann M., Iyyer M., Gardner M., Clark C., Lee K., Zettlemoyer L. (2018). Deep Contextualized Word Representations. Proceedings of the 2018 Conference of the North American Chapter of the Association for Computational Linguistics: Human Language Technologies, Volume 1 (Long Papers).

[24] Brown T., Mann B., Ryder N., Subbiah M., Kaplan J.D. (2020). Language Models are Few-Shot Learners. Proceedings of the Advances in Neural Information Processing Systems.

[25] Liu Y., Ott M., Goyal N., Du J., Joshi M., Chen D., Levy O., Lewis M., Zettlemoyer L., Stoyanov V. (2019). Roberta: A robustly optimized bert pretraining approach. arXiv.

[26] Albawi S., Mohammed T.A., Al-Zawi S. (2017). Understanding of a convolutional neural network. Proceedings of the 2017 International Conference on Engineering and Technology (ICET).

[27] Ren S., He K., Girshick R., Sun J. (2015). Faster R-CNN: Towards real-time object detection with region proposal networks. Advances in Neural Information Processing Systems.

[28] He K., Zhang X., Ren S., Sun J. Deep residual learning for image recognition. Proceedings of the IEEE Conference on Computer Vision and Pattern Recognition.

[29] Nojavanasghari B., Gopinath D., Koushik J., Baltrušaitis T., Morency L.P. Deep multimodal fusion for persuasiveness prediction. Proceedings of the 18th ACM International Conference on Multimodal Interaction.

[30] Zadeh A., Liang P.P., Mazumder N., Poria S., Cambria E., Morency L.P. Memory fusion network for multi-view sequential learning. Proceedings of the AAAI Conference on Artificial Intelligence.

[31] Li R., Wu X., Li A., Wang M. (2022). HFBSurv: Hierarchical multimodal fusion with factorized bilinear models for cancer survival prediction. Bioinformatics.

[32] Pruthi D., Gupta M., Dhingra B., Neubig G., Lipton Z.C. (2020). Learning to Deceive with Attention-Based Explanations. Proceedings of the 58th Annual Meeting of the Association for Computational Linguistics.

[33] Liu Z., Shen Y., Lakshminarasimhan V.B., Liang P.P., Bagher Zadeh A., Morency L.P. (2018). Efficient Low-rank Multimodal Fusion With Modality-Specific Factors. Proceedings of the 56th Annual Meeting of the Association for Computational Linguistics (Volume 1: Long Papers).

[34] Liu Y., Fan Q., Zhang S., Dong H., Funkhouser T., Yi L. Contrastive Multimodal Fusion With TupleInfoNCE. Proceedings of the IEEE/CVF International Conference on Computer Vision (ICCV).

[35] Wang Y., Huang W., Sun F., Xu T., Rong Y., Huang J., Larochelle H., Ranzato M., Hadsell R., Balcan M.F., Lin H. (2020). Deep Multimodal Fusion by Channel Exchanging. Proceedings of the Advances in Neural Information Processing Systems.

[36] Evangelopoulos G., Zlatintsi A., Potamianos A., Maragos P., Rapantzikos K., Skoumas G., Avrithis Y. (2013). Multimodal saliency and fusion for movie summarization based on aural, visual, and textual attention. IEEE Trans. Multimed..

[37] Li H., Zhu J., Ma C., Zhang J., Zong C. Multi-modal summarization for asynchronous collection of text, image, audio and video. Proceedings of the 2017 Conference on Empirical Methods in Natural Language Processing.

[38] Sanabria M., Precioso F., Menguy T. A deep architecture for multimodal summarization of soccer games. Proceedings of the 2nd International Workshop on Multimedia Content Analysis in Sports.

[39] Zhu J., Li H., Liu T., Zhou Y., Zhang J. MSMO: Multimodal Summarization with Multimodal Output. Proceedings of the EMNLP.

[40] Li H., Zhu J., Zhang J., He X., Zong C. (2020). Multimodal Sentence Summarization via Multimodal Selective Encoding. Proceedings of the 28th International Conference on Computational Linguistics.

[41] Zhu J., Xiang L., Zhou Y., Zhang J., Zong C. (2021). Graph-based Multimodal Ranking Models for Multimodal Summarization. Trans. Asian Low-Resour. Lang. Inf. Process..

[42] Gutmann M., Hyvärinen A. Noise-contrastive estimation: A new estimation principle for unnormalized statistical models. Proceedings of the Thirteenth International Conference on Artificial Intelligence and Statistics. JMLR Workshop and Conference Proceedings.

[43] Oord A.v.d., Li Y., Vinyals O. (2018). Representation learning with contrastive predictive coding. arXiv.

[44] He K., Fan H., Wu Y., Xie S., Girshick R. Momentum contrast for unsupervised visual representation learning. Proceedings of the IEEE/CVF Conference on Computer Vision and Pattern Recognition.

[45] Chen X., Fan H., Girshick R., He K. (2020). Improved baselines with momentum contrastive learning. arXiv.

[46] Chen T., Kornblith S., Norouzi M., Hinton G. (2020). A Simple Framework for Contrastive Learning of Visual Representations. arXiv.

[47] Yan Y., Li R., Wang S., Zhang F., Wu W., Xu W. (2021). ConSERT: A Contrastive Framework for Self-Supervised Sentence Representation Transfer. Proceedings of the 59th Annual Meeting of the Association for Computational Linguistics and the 11th International Joint Conference on Natural Language Processing (Volume 1: Long Papers).

[48] Gao T., Yao X., Chen D. SimCSE: Simple Contrastive Learning of Sentence Embeddings. Proceedings of the Empirical Methods in Natural Language Processing (EMNLP).

[49] Li X., Yin X., Li C., Zhang P., Hu X., Zhang L., Wang L., Hu H., Dong L., Wei F. (2020). Oscar: Object-semantics aligned pre-training for vision-language tasks. Proceedings of the European Conference on Computer Vision.

[50] Lei J., Li L., Zhou L., Gan Z., Berg T.L., Bansal M., Liu J. Less is more: Clipbert for video-and-language learning via sparse sampling. Proceedings of the IEEE/CVF Conference on Computer Vision and Pattern Recognition.

[51] Yuan X., Lin Z., Kuen J., Zhang J., Wang Y., Maire M., Kale A., Faieta B. Multimodal contrastive training for visual representation learning. Proceedings of the IEEE/CVF Conference on Computer Vision and Pattern Recognition.

[52] Miech A., Alayrac J.B., Smaira L., Laptev I., Sivic J., Zisserman A. End-to-end learning of visual representations from uncurated instructional videos. Proceedings of the IEEE/CVF Conference on Computer Vision and Pattern Recognition.

[53] Ba J.L., Kiros J.R., Hinton G.E. (2016). Layer normalization. arXiv.

[54] Qu L., Liu M., Cao D., Nie L., Tian Q. Context-aware multi-view summarization network for image-text matching. Proceedings of the 28th ACM International Conference on Multimedia.

[55] Li H., Zhu J., Liu T., Zhang J., Zong C. Multi-modal Sentence Summarization with Modality Attention and Image Filtering. Proceedings of the IJCAI.

[56] Reimers N., Gurevych I. (2019). Sentence-BERT: Sentence Embeddings using Siamese BERT-Networks. EMNLP-IJCNLP.

[57] Lin C.Y. ROUGE: A Package for Automatic Evaluation of Summaries. Proceedings of the Text Summarization Branches Out.

[58] Liu C.W., Lowe R., Serban I., Noseworthy M., Charlin L., Pineau J. (2016). How NOT To Evaluate Your Dialogue System: An Empirical Study of Unsupervised Evaluation Metrics for Dialogue Response Generation. Proceedings of the 2016 Conference on Empirical Methods in Natural Language Processing.

[59] Erkan G., Radev D.R. (2004). Lexrank: Graph-based lexical centrality as salience in text summarization. J. Artif. Intell. Res..

[60] Ha T.T., Nguyen T.C., Nguyen K.H., Vu V.C., Nguyen K.A. (2018). Unsupervised Sentence Embeddings for Answer Summarization in Non-factoid CQA. Comput. Sist..

[61] See A., Liu P.J., Manning C.D. (2017). Get To The Point: Summarization with Pointer-Generator Networks. Proceedings of the 55th Annual Meeting of the Association for Computational Linguistics (Volume 1: Long Papers).

[62] Li Z., Peng Z., Tang S., Zhang C., Ma H. (2020). Text Summarization Method Based on Double Attention Pointer Network. IEEE Access.

